# Supplementary material for: Task-based activation and resting-state connectivity predict individual differences in semantic capacity for complex semantic knowledge
Source: Commun Biol. 2023 Oct 9;6:1020. doi: 10.1038/s42003-023-05400-1 (PMC10562439; doi:10.1038/s42003-023-05400-1)
Supplement: Supplementary file 2 — Supplementary Information [file 42003_2023_5400_MOESM2_ESM.pdf]

Table S1. Question stimuli used in experiment in Italian and English.

Person Knowledge	
Quale filosofo ha pronunciato la frase 'So di non sapere'?	Which philosopher uttered the phrase 'I know I don't know'?
Qual é il nome dell'attore protagonista del film 'Il gladiatore'?	What is the name of the leading actor of the film 'The gladiator'?
Qual è il nome dello storico chitarrista dei Queen?	What is the name of the historic Queen guitarist?
Qual è il nome dell'attuale sindaco della cittá di Roma?	What is the name of the current mayor of the city of Rome?
Qual é il nome dell'attuale ministro italiano per gli affari esteri?	What is the name of the current Italian Minister for Foreign Affairs?
Come si chiama il protagonista del film di Hitchcock, 'Psycho'?	What is the name of the protagonist of the Hitchcock movie, 'Psycho'?
Qual è il secondo nome di Ciampi, ex presidente della Repubblica?	What is the middle name of Ciampi, former president of the Republic?
Chi è stato il primo James Bond nella storia del cinema?	Who was the first James Bond in the history of cinema?
Chi è l'attore che interpreta Tony Montana in 'Scarface'?	Who is the actor playing Tony Montana in 'Scarface'?
Chi ha diretto e interpretato il film 'Viaggi di nozze'?	Who directed and starred in the movie 'Honeymoons'?
Chi é lo scrittore francese del romanzo 'I miserabili'?	Who is the French writer of the novel 'The Miserable'?
Qual è il nome dell'attrice protagonista di 'Pretty Woman'?	What is the name of the lead actress of 'Pretty Woman'?
Qual è il nome dell'attuale primo ministro del Canada?	What is the name of the current prime minister of Canada?
Con chi è stata fidanzata Gwyneth Paltrow negli anni 90?	Who was Gwyneth Paltrow dating in the 90s?
Come si chiama l'alterego di Anakin Skywalker in 'Star Wars'?	What is the name of Anakin Skywalker's alter ego in 'Star Wars'?
Qual è il nome del famoso poeta Ottocentesco nato a Recanati?	What is the name of the famous nineteenth-century poet born in Recanati?
Qual è il nome dell'attrice che ha interpretato 'Il Cigno Nero'?	What is the name of the actress who played 'The Black Swan'?
Qual è il cognome dello statista italiano Camillo conte di Cavour?	What is the surname of the Italian statesman Camillo Count of Cavour?
Qual è il nome proprio del grande poeta Montale?	What is the proper name of the great poet Montale?
Chi è stato il pittore impressionista che dipingeva spesso ballerine?	Who was the impressionist painter who often painted dancers?
Qual è il nome dell'attore protagonista de 'La dolce vita'?	What is the name of the leading actor of 'La dolce vita'?
Come si chiama l'attore che ha interpretato il celebre Fantozzi?	What is the name of the actor who played the famous Fantozzi?
Come si chiama il protagonista de 'Il Signore degli Anelli'?	What is the name of the protagonist of 'The Lord of the Rings'?
Come si chiama il famoso critico d'arte ed ex parlamentare?	What is the name of the famous art critic and former parliamentarian?
Come si chiamava l'amante piú famosa di Benito Mussolini?	What was the name of Benito Mussolini's most famous lover?

Chi é l'autore del romanzo ottocentesco 'I tre moschettieri'?	Who is the author of the nineteenth-century novel 'The Three Musketeers'?
Chi ha scritto molti dei testi delle canzoni di Lucio Battisti?	Who wrote many of the lyrics of Lucio Battisti's songs?
Chi è la politica e più celebre nipote di Sophia Loren?	Who is the politician and most famous granddaughter of Sophia Loren?
Quale famoso pittore del quattrocento ha affrescato la cappella Sistina?	Which famous painter of the fifteenth century frescoed the Sistine chapel?
Qual è il nome dell'attore che ha impersonato il 'Grinch'?	What is the name of the actor who played the 'Grinch'?
Chi è lo storico conduttore del programma 'Chi vuol esser milionario'?	Who is the historic host of the 'Who Wants to Be a Millionaire' program?
Come si chiama Paolo Kessisoglu nella sit-com 'Camera Cafè'?	What is the name of Paolo Kessisoglu in the sitcom 'Camera Cafè'?
Chi é la prima ginnasta olimpionica rumena ad aver ottenuto dieci?	Who is the first Romanian Olympic gymnast to have scored ten?
Qual era il nome dello storico cantante dei Queen?	What was the name of the historic Queen singer?
Qual é il nome del pittore, fondatore del cubismo?	What is the name of the painter, founder of Cubism?
Come si chiama l'attore che ha interpretato 'Forrest Gump'?	What is the name of the actor who played 'Forrest Gump'?
Nel romanzo, qual é il vero nome del Conte di Montecristo?	In the novel, what is the real name of the Count of Monte Cristo?
Qual è il nome completo dello scorso Presidente della Repubblica?	What is the full name of the last President of the Republic?
Qual é il vero nome di Superman da borghese?	What is Superman's real name as a bourgeois?
Qual é il nome del primo ministro della Germania?	What is the name of the prime minister of Germany?
Nel fumetto, chi è la compagna di vita di Diabolik?	In the comic, who is Diabolik's life partner?
Quale é il nome del conduttore storico di 'Zelig'?	What is the name of the historical conductor of 'Zelig'?
Come si chiama la miglior amica di Harry Potter?	What is the name of Harry Potter's best friend?
Quale attore interpreta Nando Moriconi in 'Un americano a Roma'?	Which actor plays Nando Moriconi in 'An American in Rome'?
Come si chiama lo scrittore triestino de 'La coscienza di Zeno'?	What is the name of the Trieste writer of 'Zeno's conscience'?
Chi è la modella con cui era fidanzato Johnny Depp?	Who is the model Johnny Depp was engaged to?
Qual è il nome dell'attore protagonista di 'Gran Torino'?	What is the name of the leading actor of 'Gran Torino'?
Chi è la top model, prima moglie di Richard Gere?	Who is the supermodel, Richard Gere's first wife?
Come si chiamava la scrittrice britannica di 'Gita al faro'?	What was the British writer of 'Trip to the Lighthouse' called?
Come si chiama il famoso tennista svizzero, numero tre nel mondo?	What is the name of the famous Swiss tennis player, number three in the world?
Come si chiama in arte il cantante Lorenzo Cherubini?	What is the singer Lorenzo Cherubini called in art?
Come si chiama l'autore del programma "Striscia la notizia"?	What is the name of the author of the program "Striscia la Notizia"?
Chi è la comica trasgressiva di 'Che Tempo che Fa'?	Who is the transgressive comedian of 'Che Tempo che Fa'?

Qual era il secondo nome dello scrittore del mistero Edgar Poe?
Come si chiama l'attrice premio oscar de 'Il diavolo veste Prada'?
Come si chiama la pittrice messicana nota per i suoi autoritratti?
Qual è il nome dello scrittore novecentesco de 'La Metamorfosi'?
Qual è il nome di Spider-Man quando é in borghese?
Come si chiama l'attore protagonista del film 'La vita è bella'?
Chi era l'artista che cantava 'Cuore matto', negli anni 60?

Place Knowledge

Quale città della Basilicata è anche conosciuta come "La città dei Sassi"?
Come si chiama il monumento torinese col museo del cinema?
Se sto osservando il Cristo Redentore, in quale stato mi trovo?
Come si chiama il capoluogo della regione Calabria?
Quale isola siciliana è l'estremità italiana più a sud?
Se mi trovo a Budapest, in quale nazione sono?
Qual é il nome della montagna principale della Tanzania?
Come si chiama il lago che bagna Uganda, Kenya e Tanzania ?
In quale città si trova il memoriale dedicato al presidente Lincoln?
Quale tra i maggiori fiumi del mondo ha origine dall'Himalaya?
In quale nazione si trova la fortezza di Machu Picchu?
Come si chiama il principale fiume che bagna l'Africa settentrionale?
Se mi trovo a passeggiare a Nantes, in quale stato sono?
Quale rione di Roma è famoso per la sua atmosfera bohémien?
Qual é il mausoleo indiano costruito in memoria dell'imperatrice?
Qual é il nome della cittá, capitale dell'Australia?
Se mi trovo a Bruxelles, in quale nazione mi trovo?
Dove dovresti andare per ammirare la Cappella degli Scrovegni?
In quale città spagnola si trova il complesso di Alhambra?

What was the middle name of mystery writer Edgar Poe?
What is the name of the Oscar-winning actress of 'The devil wears Prada'?
What is the name of the Mexican painter known for her self-portraits?
What is the name of the twentieth-century writer of 'La Metamorphosis'?
What's Spider-Man's name when he's in plain clothes?
What is the name of the lead actor of the film 'Life is beautiful'?
Who was the artist who sang 'Cuore matto', in the 60s?

Which city of Basilicata is also known as "The city of the Sassi"?
What is the name of the Turin monument with the cinema museum?
If I am observing Christ the Redeemer, what state am I in?
What is the name of the capital of the Calabria region?
Which Sicilian island is the southernmost tip of Italy?
If I am in Budapest, which country am I in?
What is the name of the main mountain in Tanzania?
What is the name of the lake that bathes Uganda, Kenya and Tanzania?
In which city is the memorial dedicated to President Lincoln located?
Which of the major rivers in the world originates from the Himalayas?
In which country is the fortress of Machu Picchu located?
What is the name of the main river that flows through northern Africa?
If I am walking around Nantes, what state am I in?
Which neighborhood in Rome is famous for its bohemian vibe?
What is the Indian mausoleum built in memory of the empress?
What is the name of the city, capital of Australia?
If I am in Brussels, which country am I in?
Where should you go to see the Scrovegni Chapel?
In which Spanish city is the Alhambra complex located?

Quale mare bagna le coste occidentali della Corea del Sud?	Which sea washes the western coasts of South Korea?
Come si chiama il canale tra Mar Rosso e Mar Mediterraneo?	What is the name of the channel between the Red Sea and the Mediterranean Sea?
Qual é il nome della capitale dello stato dell'Afghanistan?	What is the name of the state capital of Afghanistan?
Da quale mare sono bagnate le coste della Polonia?	From which sea are the coasts of Poland washed?
Qual è la più grande città statunitense più vicina a Cuba?	What is the largest US city closest to Cuba?
Quale sito archeologico inglese crea un insieme circolare di megaliti?	Which English archaeological site creates a circular ensemble of megaliths?
Se mi trovo in Cappadocia, in quale nazione sono?	If I am in Cappadocia, which country am I in?
Qual è il nome del principale fiume che scorre a Praga?	What is the name of the main river that flows in Prague?
Quale cittá è il capoluogo di provincia dell'Isola d'Elba?	Which city is the provincial capital of the Island of Elba?
In quale città si trova la famosa Porta di Brandeburgo?	In which city is the famous Brandenburg Gate located?
Quale nazione funge da confine settentrionale tra Algeria e Libia?	Which nation serves as the northern border between Algeria and Libya?
Quale arcipelago è conosciuto come il Commonwealth delle Indie Orientali?	Which archipelago is known as the Commonwealth of the East Indies?
Quale é il fiume che scorre tra Thailandia, Laos e Birmania?	What is the river that flows between Thailand, Laos and Burma?
Come si chiama la grande torre dell'orologio di Londra?	What is the name of the great clock tower in London?
Se mi trovo ad Hanoi, sono in quale nazione orientale?	If I am in Hanoi, am I in which eastern country?
Di quale isole fa parte la famosa meta turistica Ibiza?	Which islands does the famous tourist destination Ibiza belong to?
Quale stato africano é bagnato maggiormente dal golfo di Guinea?	Which African country is wet most by the Gulf of Guinea?
Qual é il fiume di confine tra Veneto ed Emilia-Romagna?	What is the border river between Veneto and Emilia-Romagna?
Come si chiama il lago che bagna la città di Ginevra?	What is the name of the lake that bathes the city of Geneva?
Qual è il fiume principale che attraversa la metropoli di Parigi?	What is the main river that flows through the metropolis of Paris?
Come si chiama il capoluogo del Friuli Venezia Giulia?	What is the name of the capital of Friuli Venezia Giulia?
Come si chiama la chiesa dietro la scalinata di Piazza di Spagna?	What is the name of the church behind the Spanish Steps?
In quale stato americano si trova il casinò Il Bellagio?	In which US state is Il Bellagio casino located?
Quale città italiana ospita al suo interno una corsa di cavalli?	Which Italian city hosts a horse race inside?
Se mi trovo a Tripoli, sono nella capitale di quale nazione?	If I am in Tripoli, I am in the capital of which country?
Se mi trovo a Damasco, in quale nazione sono?	If I am in Damascus, what country am I in?
Quale penisola ha il territorio quasi interamente coperto dalla Turchia?	Which peninsula has the territory almost entirely covered by Turkey?
Quale fiume principale nasce in Svizzera e sfocia nel Po?	Which main river originates in Switzerland and flows into the Po?

Come si chiamano le grandi pianure erbose situate in Argentina?
In quale città europea si trova il palazzo di Schonbrunn?
Quale lago bagna le coste di Perù e Bolivia?
Qual é la cittá che fa da capitale al Brasile?
Come si chiama la famosa basilica cattolica situata a Barcellona?
Come si chiama il fiume che attraversa la città di Firenze?
Come si chiama il principale fiume che attraversa Roma?
Qual é il nome del capoluogo della regione Basilicata?
Come si chiama la capitale delle famose isole tropicali Hawaii?
Qual é il mare che bagna anche lo stato dell'Ucraina?
Come si chiama la più famosa città Santa dell'Arabia Saudita?
Come si chiama il tempio greco situato nell'acropoli di Atene?
Se mi trovo a Piazza San Marco, in quale città sono?

What are the great grassy plains located in Argentina called?
In which European city is Schonbrunn Palace located?
Which lake bathes the coasts of Peru and Bolivia?
What is the city that is the capital of Brazil?
What is the name of the famous Catholic basilica located in Barcelona?
What is the name of the river that crosses the city of Florence?
What is the name of the main river that crosses Rome?
What is the name of the capital of the Basilicata region?
What is the capital of the famous tropical Hawaiian islands called?
What is the sea that also bathes the state of Ukraine?
What is the name of the most famous holy city in Saudi Arabia?
What is the name of the Greek temple located in the Acropolis of Athens?
If I am in Piazza San Marco, which city am I in?

Object Knowledge

Come si chiama l'elemento portante che scarica il peso sulle colonne?
Come si chiama l'utensile solitamente utilizzato per raccogliere le foglie?
Come si chiama l'utensile per lisciare il legno?
Quale lente permette di correggere la vista di un solo occhio?
Come si chiama la ceramica smaltata con argilla per fare i vasi?
Come si chiama la parte del fucile utilizzata come impugnatura?
Come si chiama il dispositivo di sicurezza interno al volante di un'auto?
Come si chiama la memoria temporanea di un computer?
Qual é lo strumento che serve per afferrare i chiodi?
Quale antica arte giapponese crea figure da fogli di carta?
Come si chiama l'elemento che collega il pedale alla bici?
Quale oggetto di cancelleria serve ad appuntire le matite?
Come è chiamata una zuppiera o insalatiera di ceramica?

What is the name of the load-bearing element that unloads the weight on the columns?
What is the name of the tool usually used to collect the leaves?
What is the name of the tool for smoothing wood?
Which lens allows you to correct the vision of only one eye?
What is the name of the glazed pottery with clay used to make the vases?
What is the name of the part of the rifle used as the handle?
What is the name of the internal safety device behind the wheel of a car?
What is the temporary memory of a computer called?
What is the tool you need to grab nails?
What ancient Japanese art creates figures from sheets of paper?
What is the name of the element that connects the pedal to the bike?
Which stationery object is used to sharpen the pencils?
What is a ceramic bowl or salad bowl called?

Qual è l'attrezzo piú usato per l'allenamento indoor nel canottaggio?

Quale chiave meccanica è caratterizzata da una sezione esagonale?

Quale indumento femminile é composto da un laccio di stoffa?

Quale dispositivo permette di azionare il fanale della bicicletta?

Come si chiama il foglio ricavato da una pianta acquatica?

Di che materiale è fatta la palla da polo?

Come viene anche definito un balcone o parapetto esterno continuo?

Come si chiama l'elemento di raccodo tra colonna ed architrave?

Come si chiama l'antico strumento per la misurazione del tempo?

Qual è l'accessorio che si indossa per spronare il cavallo?

Quale materiale costituisce principalmente le batterie dei cellulari?

Come si chiama lo strumento per la misurazione degli angoli?

Quale oggetto serve a proteggersi le dita mentre si cuce?

Quale oggetto sferico o tondeggiante è posto all'esterno di una porta?

Come si chiama lo strumento di misurazione istantanea della velocità?

Come si chiama la parte del tetto da dove esce il fumo?

Come si chiama lo strumento che misura la pressione sanguigna?

Di quale materiale sono fatte le pietre del curling?

Qual è l'oggetto di illuminazione portatile a forma di gabbia?

Come si chiama il risvolto dei cappotti che circonda il collo?

Come si chiama il dispositivo che cambia la temperatura interna dell'auto?

Come si chiama l'antico strumento per il calcolo matematico?

Qual é lo strumento che permette di manovrare un kayak?

Come si chiama il macchinario utilizzato per produrre i tessuti?

Qual è l'attrezzo, usato in ginnastica ritmica, simile al birillo?

Come si chiama l'oggetto usato nel gioco del Badmington?

Quale strumento d'ufficio permette la veloce rimozione delle graffette?

Qual é la parte della chitarra dove sono agganciate le corde?

What is the most used equipment for indoor rowing training?

Which mechanical key has a hexagonal section?

Which women's garment is made up of a fabric lace?

Which device allows you to operate the bicycle light?

What is the name of the sheet obtained from an aquatic plant?

What material is the polo ball made of?

How is a continuous external balcony or parapet also defined?

What is the name of the connecting element between column and lintel?

What is the name of the ancient instrument for measuring time?

What is the accessory you wear to spur the horse?

What material mainly makes up cellphone batteries?

What is the name of the angle measuring tool?

What object is used to protect the fingers while sewing?

What spherical or rounded object is placed on the outside of a door?

What is the name of the instant speed measurement tool?

What is the name of the part of the roof where the smoke comes out?

What is the name of the instrument that measures blood pressure?

What material are curling stones made of?

What is the portable cage-shaped lighting object?

What is the name of the lapel of the coats that surrounds the neck?

What is the name of the device that changes the car's interior temperature?

What is the name of the ancient tool for mathematical calculation?

What is the tool that allows you to maneuver a kayak?

What is the name of the machinery used to produce the fabrics?

What is the tool, used in rhythmic gymnastics, similar to the pin?

What is the name of the object used in the Badmington game?

Which office tool allows quick removal of staples?

What is the part of the guitar where the strings are hooked?

Qual é il nome del vetro anteriore delle auto?	What is the name of the front glass of cars?
Qual é l'arma che scaglia frecce grazie ad un grilletto?	What is the weapon that shoots arrows with a trigger?
Come si chiama lo strumento da disegno a forma di triangolo?	What is the name of the triangle drawing tool?
Come si chiamano i tappeti da parete con raffigurazioni artistiche?	What are the name of the wall rugs with artistic representations?
Di che materiale é fatta la lavagna di tipo classico?	What material is the classic type blackboard made of?
Come si chiama il macchinario per il taglio di salumi?	What is the name of the sausage cutting machine?
Qual é la parte della tromba dove si appoggiano le labbra?	What is the part of the trumpet where the lips rest?
Di quale famiglia di strumenti musicali fa parte il violino?	Which family of musical instruments does the violin belong to?
Come si chiama la penna dotata di serbatoio ricaricabile per l'inchiostro?	What is the name of the pen with refillable ink reservoir?
Come si chiama il pugnale posto sulla canna del fucile?	What is the name of the dagger placed on the barrel of the gun?
Come è chiamata la riproduzione cartografica dell'intera superficie terrestre?	What is the cartographic reproduction of the entire earth's surface called?
Quale dispositivo meccanico permette di girare agilmente il volante?	Which mechanical device allows you to easily turn the steering wheel?
Qual è lo strumento che misura l'intensità della corrente elettrica?	What is the instrument that measures the intensity of the electric current?
Da cosa è rivestita la palla utilizzata nel tennis?	What is the ball used in tennis coated with?
Di che materiale sono composti solitamente i bolognini nelle strade?	What material are Bologninis usually made of in the streets?
Qual é l'utensile in legno sul quale si affettano gli alimenti?	What is the wooden utensil on which food is sliced?
Quale tipo di pentola viene usata tradizionalmente per preparare la polenta?	What type of pot is traditionally used to prepare polenta?
Qual é lo strumento in grado di misurare la pressione atmosferica?	What is the instrument capable of measuring atmospheric pressure?
Quale strumento da laboratorio si usa per prelevare quantità definite di liquido?	Which laboratory instrument is used to withdraw defined quantities of liquid?

Scholastic Knowledge

Come si chiama la scienza che studia la crosta terrestre?	What is the name of the science that studies the earth's crust?
Qual è l'unità di misura americana equivalente al chilometro?	What is the American unit of measurement equivalent to the kilometer?
Qual é l'antica forma di scambio di beni senza uso di moneta?	What is the ancient form of exchange of goods without the use of money?
Come si chiama l'Era compresa tra Paleozoico e Cenozoico?	What is the name of the Era between Paleozoic and Cenozoic?
Come si chiama l'ordine animale appartengono gli umani?	What does the animal order belong to humans?
Quale aggettivo verbale latino esprime dovere o necessitá?	Which Latin verbal adjective expresses duty or necessity?
Come è detto qualcuno che cambia idea facilmente?	How do you say someone who changes their mind easily?

Come si chiama il caso grammaticale del complemento di termine?
Di chi sono le leggi spiegano il movimento dei pianeti?
Come è definito nella musica classica un andamento veloce e gioioso?
Quale area ottieni con: diagonale minore per diagonale maggiore diviso due?
Contro chi si scontrarono i romani nella II Guerra Punica?
Di quale popolo fu dittatore il terribile generale Pinochet?
Qual è la scienza che studia il rapporto tra ambiente e viventi?
Come viene comunemente definito un materiale resistente all'acqua?
Con quale teorema geometrico si calcola la misura dell'ipotenusa?
Qual é il nome della scienza che studia le cavità sotterranee?
Che forza contrasta lo spostamento di un corpo su una superficie?
Quale impero turco ha resistito nell'europa orientale per oltre sei secoli?
Come è definito il processo di ipotermia controllata?
Come viene definito lo studio della validità del ragionamento?
Come si chiama il periodo storico che precede la scrittura?
Quale movimento della Terra attorno al Sole segue un’orbita ellittica?
Come si chiama il passaggio della materia dallo stato solida a gassoso?
Come puó essere anche definito il complemento diretto?
Qual è il nome del piccolo corpo celeste simile ad un pianeta?
Come si chiama una raccolta scelta di opere letterarie?
Qual é il punto di massima distanza di un pianeta dal Sole?
Come si chiama la patologia caratterizzata da continue cefalee?
Qual é il nome dell'unitá di misura della potenza?
Come è chiamata la tavola degli elementi in chimica?
Quale nome si usa per indicare l'arte della persuasione tramite il linguaggio?
Come è chiamata la scienza che studia il comportamento animale?
Oltre alle basi cosa serve per calcolare l'area del trapezio?
Come si chiama l'atto di disobbedienza della ciurma?

What is the grammatical case of the term complement called?
Whose laws explain the movement of the planets?
How is a fast and joyful movement defined in classical music?
Which area do you get with: minor diagonal by major diagonal divided by two?
Against whom did the Romans clash in the Second Punic War?
Of which people was the terrible general Pinochet dictator?
What is the science that studies the relationship between the environment and the living?
How is a water resistant material commonly defined?
With which geometric theorem is the measure of the hypotenuse calculated?
What is the name of the science that studies underground cavities?
What force contrasts the movement of a body on a surface?
Which turkish empire stood in eastern europe for over six centuries?
How is the controlled hypothermia process defined?
How is the study of the validity of reasoning defined?
What is the name of the historical period that precedes the writing?
What movement of the Earth around the Sun follows an elliptical orbit?
What is the name of the passage of matter from a solid to a gaseous state?
How can the direct complement also be defined?
What is the name of the small, planet-like celestial body?
What is the name of a select collection of literary works?
What is the maximum distance of a planet from the Sun?
What is the name of the disease characterized by continuous headaches?
What is the name of the unit of measurement of power?
What is the table of elements called in chemistry?
What name is used to indicate the art of persuasion through language?
What is the science that studies animal behavior called?
In addition to the basics, what do you need to calculate the area of the trapezoid?
What is the crew's act of disobedience called?

Quale movimento politico settecentesco si fondava sulla ragione?	What eighteenth-century political movement was based on reason?
Come si indica la vendita concentrata nelle mani di un solo venditore?	How do you indicate concentrated sales in the hands of a single seller?
Quale unità di misura indica l'intensità di flusso magnetico?	Which unit of measurement indicates the intensity of magnetic flux?
Come si chiama l'applicazione dell'informatica all'automazione di dispositivi domestici?	What is the application of information technology to the automation of home devices called?
In quale regime politico il potere è nelle mani di poche persone?	In what political regime is power in the hands of a few people?
Come si chiama la figura retoria che accosta due parole opposte?	What is the name of the rhetorical figure who juxtaposes two opposite words?
Come si chiama il processo di formazione di qualunque rilievo montuoso?	What is the formation process of any mountain relief called?
Come si definisce il tasso di cambiamento di una funzione matematica?	How do you define the rate of change of a mathematical function?
Per ottenere la media aritmetica qual é la prima operazione da fare?	To obtain the arithmetic mean what is the first operation to do?
Come si chiama il tessuto che riveste le ossa articolari?	What is the name of the tissue that lines the joint bones?
Come si chiama un triangolo con due lati uguali?	What is a triangle with two equal sides called?
Qual è la figura retorica basata su similitudini sottintese?	What is the rhetorical figure based on implied similarities?
Come si chiama l'abbandono volontario del potere da parte di un sovrano?	What is the voluntary abandonment of power by a sovereign called?
Come si chiama la ghiandola endocrina alla base del cranio?	What is the endocrine gland at the base of the skull called?
Qual é la materia che studia i triangoli a partire dagli angoli?	What is the subject that studies triangles starting from the angles?
A quale popolo antico é attribuita l'invenzione della ruota?	To which ancient people is the invention of the wheel attributed?
Come si chiama la disciplina che si concentra sullo studio dei testi?	What is the name of the discipline that focuses on the study of texts?
In chimica, qual é il contrario di un acido?	In chemistry, what is the opposite of an acid?
In quale modo si chiama l’acido comunemente chiamato DNA?	What is the name of the acid commonly called DNA?
Qual é il movimento letterario ottocentesco contrario al romanticismo italiano?	What is the nineteenth-century literary movement opposed to Italian romanticism?
Come si chiama la morte cellulare non reversibile, con sfaldamento della membrana?	What is the name of non-reversible cell death, with flaking of the membrane?
Come si chiama l'aumento dei prezzi, associato al minor potere di acquisto?	What is the price increase associated with lower purchasing power called?
Quale unità di misura esprime la quantità di intensità luminosa?	Which unit of measurement expresses the quantity of light intensity?
Come viene chiamata l'unitá di misura della frequenza?	What is the frequency unit called?
Come si chiama il periodo di vita latente trascorso da alcuni animali?	What is the latent period of some animals called?

Table S2. Semantic-encoding test stimuli

Italian

	Fact	Question
1.	'Dolophones conifera, é detto "ragno avvolgente" ed é un ragno indigeno dell''Australia, il quale ha la capacitá di appiattire il proprio corpo e di arrotolarsi intorno ai rami, camuffandosi alla perfezione'	'Come é definito il Dolophones conifera che puó appiattire il proprio corpo?'
2.	'Thomas Young, é stato nominato "L''Ultimo Uomo che Sapeva Tutto", ha dimostrato la natura ondulatoria della luce, ha sviluppato la teoria della capillarità e tra le altre cose decifrò dei geroglifici egizi'	'Che cosa aveva decifrato Thomas Young, "L'Ultimo Uomo che Sapeva Tutto"?'
3.	'La forzatura è un metodo per la coltivazione del rabarbaro. Per effettuarla, si trasportano le piante in un luogo buio e tenute al lume di candela. Questo metodo permette alle piante di svilupparsi oltre due centimetri al giorno'	'Come si chiama il metodo per la coltivazione del rabarbaro in cui le piante vengono portate in luogo buio?'
4.	'Nel 1904, le bustine del tè vennero inventate per sbaglio da Thomas Sullivan, che pensò che fosse più conveniente mandare dei piccoli campioni in bustine di seta. Credendo fossero da inzuppare, venne inondato di ordini per le sue "bustine da tè"'	'Cosa venne inventato per sbaglio da Thomas Sullivan nel 1904?'
5.	'In Giappone, se un giorno festivo cade di domenica, il giorno successivo diventa festa nazionale. Allo stesso modo, se un giorno feriale cade tra due festività, diventerà festa a sua volta. In questo caso, il giorno prende il nome di "festa dei cittadini'	'Come é definito in Giappone un giorno feriale che diventa festivo, se cade tra due festivitá?'
6.	'Una cittadina rurale giapponese, si trova attualmente sotto assedio da parte delle scimmie. La cittadina è difesa da tre donne anziane armate di fucili ad aria. Le intrepide anziane si fanno chiamare le "Monkey Busters"'	'Chi sono le "Monkey Busters", armate di fucili ad aria?'
7.	' Esiste una rara alga verde, chiamata Marimo, che si sviluppa in palle morbide e vellutate. Trovate principalmente nel lago Akan in Giappone, queste palle possono avere un diametro di 20-30 centimetri'	'Come si chiama l'alga verde giapponese a forma di palla morbida e vellutata?'
8.	'Gli antichi greci e romani usavano lo zafferano come profumo. In Cina esso veniva utilizzato come medicina. Ci vogliono quasi 17.000 fiori, che vengono colti a mano, per ottenere un singolo etto di zafferano. È per questo che costa così tanto'	'Come veniva usato in Cina lo zafferano, i cui fiori vengono colti a mano?'
9.	'Nel mondo esistono oltre 7.500 cultivar di mele (Malus pumila). Il numero di varietà di mele è talmente elevato che, se una persona ne potesse assaggiare una al giorno, ci metterebbe oltre 20 anni a provarle tutte'	'Quanti anni ci vorrebbero per assaggiare un tipo di mela al giorno delle 7500 cultivar al mondo?'
10.	'Il castello di Eltz, è un castello medievale. È di proprietà della stessa famiglia da ben 33 generazioni. Si tratta di un ganerburg, cioé di un castello appartenente a una comunità di eredi e suddiviso in più famiglie'	'Da quante generazioni il castello medievale di Eltz è di proprietá della stessa famiglia?'
11.	'Un ufficiale britannico, un giorno che era pesantemente ubriaco, ha pensato che un albero baniano si muovesse verso di lui. Allora ordinó al suo sergente di arrestarlo. Fino ad allora l''albero è rimasto in catene'	'Chi ha ridotto in catene un albero baniano?'
12.	'Il geco pigmeo brasiliano ha una lunghezza appena superiore ai 2 cm e possiede una pelle idrofobica, senza la quale rischierebbe di annegare con facilità in una pozzanghera. La sua pelle unica gli permette addirittura di camminare sull''acqua'	'Che caratteristica ha la pelle del geco pigmeo brasiliano lungo circa 2 cm?'

13.	'Utilizzando piccoli microfoni sospesi tra i fiori, hanno registrato i ronzii di alcune api durante l''eclissi del 2017 in Nord America. Le api sono rimaste attive e rumorose fino al momento precedente la totalità dell'eclissi, quando si sono subito zittite'	'Durante quale evento astronomico le api, registrate in nord America hanno smesso di ronzare?'
14.	'A Los Angeles c''era un''oca che viveva ad Echo Park e che aveva formato una stretta amicizia con un residente del luogo. L''oca camminava con lui in giro per il parco, e scacciava via persino cani, oche e umani che cercavano di avvicinarsi'	'In quale cittá viveva l'oca che aveva stretto amicizia con un residente del luogo?'
15.	'Sono stati scoperti i resti del serpente più grande mai esistito al mondo. Questo serpente é chiamato "titanoboa" e sembra essere vissuto 60 milioni di anni fa. L''enorme rettile pesava oltre 1000 kg ed era lungo addirittura 14 metri'	'Quanto era lungo il serpente piú grande mai esistito e chiamato "titanoboa"?'
16.	'UbiQD, un''azienda americana di manifattura dei materiali, ha sviluppato un prodotto innovativo in cui un sottile strato di nanoparticelle si inserisce in mezzo a due pannelli di vetro: creando un pannello solare trasparente utilizzabile come finestra'	'Di che materiale é fatto lo strato che viene inserito in mezzo a due pannelli di vetro dall'azienda americana UbiQD?'
17.	'Nei primi anni ''80, le riviste di computer contenevano talvolta dei listati di codice che i lettori passavano ore e ore a copiare sulle proprie tastiere. In questo modo potevano ottenere un gioco o un particolare programma'	'Cosa contenevano le riviste di computer, utili ad ottenere giochi e programmi?'
18.	'La persona che inventò, tra le altre cose, il segnale di stop, William Phelps Eno, non imparò mai a guidare. Fu un uomo d''affari americano che viene ricordato per le innovazioni nel campo della sicurezza stradale'	'Cosa non ha mai imparato a fare l''inventore del segnale di stop, William Phelps Eno?'
19.	'La tecnica del "cassone di fondazione" è utilizzata per realizzare le fondamenta dei ponti. Prevede la costruzione di un muro che isoli un''area nell''acqua, da cui viene drenato tutto il liquido. La fondamenta del ponte è creata poi dentro al cassone'	' Come viene definita la tecnica utilizzata per le fondamenta dei ponti che prevede la costruzione di un muro che isoli l'area dall'acqua?'
20.	'Negli anni ''80 del 1800, molti contadini negli USA comunicavano collegando i loro telefoni alle recinzioni di filo spinato. Il processo permetteva a 20 persone di essere connesse allo stesso tempo: chiunque chiamasse, squillava il telefono di tutti'	'Quante persone potevano connettersi contemporaneamente collegando i propri telefoni al filo spinato nel 1880?'
21.	' L'acquaponica combina l'allevamento di pesci con la coltivazione di piante, usando l'acqua e gli scarti dei pesci per nutrire le piante. Può richiedere anche solo il 2% dell'acqua usata nei metodi standard di coltivazione'	'Con cosa combina l'allevamento di pesci, l'acquaponica, richiedendo solo il 2% di acqua?'
22.	'Un fisico di Harvard ha sviluppato una tecnologia che prende il carbonio dall'atmosfera e lo combina con l'idrogeno per creare un carburante pulito. Esso può essere usato per motori a diesel, sia per le auto che per gli aerei'	'Con cosa è combinato, oltre all'idrogeno, il carburante pulito sviluppato da un fisico di Harvard?'
23.	'Un composto chimico allucinogeno ha rimosso i sintomi di diversi pazienti depressi nel Regno Unito. Sono stati indotti viaggi psichedelici in 12 persone usandone consistenti dosi: tutti i volontari hanno mostrato notevoli segni di miglioramento'	'Dove è stato somministrato il composto chimico allucinogeno a pazienti depressi?'
24.	'Sono stati portati dei semi di ciliegio sulla Stazione Spaziale Internazionale. Dopo essere tornati sulla Terra ed essere stati piantati, gli alberi sono fioriti sei anni prima del previsto, e alcuni mostravano fiori insoliti'	'Quanti anni prima sono fioriti gli alberi di ciliegio, i cui semi erano stati sulla Stazione Spaziale Internazionale?'
25.	'Un tempo, la maggior parte delle carote era viola. Fu nel 17esimo secolo che i coltivatori olandesi scelsero di selezionare e favorire le carote arancioni, per omaggiare uno dei colori della loro bandiera dell''epoca'	'Di che colore erano la maggior parte delle carote fino al 17esimo secolo?'

English

Fact		Question
1.	‘Dolophones conifera, is called "enveloping spider" and it is an indigenous spider of Australia, which has the ability to flatten its body and to roll itself around the branches, camouflaging itself perfectly’	‘How it is the Dolophones Conifer, which can flatten one's body, defined?'
2.	'Thomas Young has been named ‘The last man who knew everything’, he demonstrated the wave nature of the light, he developed the theory of capillarity and, among other things, he deciphered Egyptian hieroglyphs’	‘What had Thomas Young, "The Last Man Who Knew Everything", deciphered?'
3.	‘”Forcing” is a method of growing rhubarb. To do this, the plants are transported to a dark place and lightened by candlelights. This method allows the plants to grow over two centimeters per day’	‘What is the name of the method for growing rhubarb, where the plants are taken to a dark place?'
4.	'In 1904, tea bags were erroneously invented by Thomas Sullivan, who thought it was more convenient to send small samples in silk bags. Believing they were to be soaked, he was inundated with orders for his "tea bags". '	‘What had Thomas Sullivan invent by mistake in 1904?'
5.	In Japan, if a public holiday falls on a Sunday, the next day becomes a national holiday. Similarly, if a weekday falls between two holidays, it will become a holiday itself. In this case, the day is called "citizens' day"	'How is a weekday that becomes a public holiday defined in Japan, if it falls between two holidays?'
6.	'A rural Japanese town is currently under siege by monkeys. The town is defended by three elderly women armed with air rifles. The intrepid elders call themselves the "Monkey Busters" '	'Who are the "Monkey Busters", whom are armed with air rifles?'
7.	‘There is a rare green alga, called Marimo, which develops into soft and velvety balls. Primarily found in Lake Akan in Japan, these balls can be 20-30 centimeters in diameter.’	'What is the name of the Japanese green alga which has the shape of a soft and velvety ball?'
8.	'The ancient Greeks and Romans used saffron as a perfume. In China it was used as a medicine. It takes almost 17,000 flowers, which are picked by hand, to obtain a single hectogram of saffron. That's why it costs so much '	'How was saffron, whose flowers are picked by hand, used in China?'
9.	‘There are over 7,500 apple cultivars (Malus pumila) worldwide. The number of apple varieties is so large that, if a person could taste one a day, it would take him over 20 years to try them all '	'How many years would it take to taste one type of apple a day, out of the 7,500 cultivars in the world?'
10.	'Eltz Castle, is a medieval castle. It has been owned by the same family for 33 generations. It is a ganerburg, that is a castle belonging to a community of heirs and divided into several families'	'How many generations has the medieval castle of Eltz been owned by the same family?'
11.	‘A British officer, who was heavily drunk one day, thought a banyan tree was moving towards him. He then ordered his sergeant to arrest him. Until then the tree remained in chains'	'Who reduced a banyan tree to chains?'
12.	'The Brazilian pygmy gecko is just over 2 cm long and has hydrophobic skin, without which it would easily drown in a puddle. His unique skin even allows him to walk on water'	'Which feature has the skin of the approximately 2 cm long Brazilian pygmy gecko?'
13.	‘Using small microphones suspended between flowers, it has been recorded the buzzing of bees during the 2017 eclipse in North America. The bees remained active and noisy until the moment before the totality of the eclipse, when they immediately fell silent '	'During what astronomical event did the bees recorded in North America stop buzzing?'

14.	In Los Angeles there was a goose who lived in Echo Park and who had formed a close friendship with a local resident. The goose walked with him around the park, and even chased away dogs, geese and humans who tried to get close '	'In which city did the goose who had befriended a local resident lived?'
15.	‘The remains of the largest snake that ever existed in the world have been discovered. This snake is called "titanoboa" and appears to have lived 60 million years ago. The huge reptile weighed over 1000 kg and was even 14 meters long’	'How long was the largest snake ever, known as “Titanoboa”?'
16.	‘UbiQD, an American materials manufacturing company, has developed an innovative product in which a thin layer of nanoparticles fits between two glass panels creating a transparent solar panel that can be used as a window'	'What material is the layer that is inserted between two glass panels, made of by the American company UbiQD?'
17.	'In the early 1980s, computer magazines sometimes contained listings of code that readers would spend countless hours copying onto their keyboards. That way they could get a game or a particular program in return'	'What did the computer magazines contain, useful for obtaining games and programs?'
18.	'The person who invented, among other things, the stop sign, William Phelps Eno, never learned to drive. He was an American businessman who is remembered for innovations in road safety'	'What did the' inventor of the stop sign, William Phelps Eno never learn to do? '
19.	'The "foundation caisson" technique is used to build the foundations of the bridges. It involves the construction of a wall that isolates an area in the water, from which all the liquid is drained. The foundation of the bridge is then created inside the caisson'	'How is the technique used for the foundations of the bridges, which involves the construction of a wall that isolates the area from the water, defined?'
20.	'In the 1880s, many farmers in the US communicated by plugging their phones into barbed wire fences. The process allowed 20 people to be connected at the same time: whoever called, everyone's phone rang'	'How many people could connect at the same time by plugging their phones into barbed wire in 1880?'
21.	'Aquaponics combines fish farming with plant cultivation, using water and fish waste to feed plants. It can require as little as 2% of the water used in standard cultivation methods'	'Con cosa combina l’allevamento di pesci, l’acquaponica, richiedendo solo il 2% di acqua?'
22.	‘A Harvard physicist has developed a technology that takes carbon from the atmosphere and combines it with hydrogen to create a clean fuel. It can be used for diesel engines, both for cars and for airplanes'	'What does fish breeding combine with the aquaponics, requiring only 2% of water?'
23.	‘A hallucinogenic chemical compound has removed the symptoms of several depressed patients in the UK. Psychedelic trips were induced in 12 people using consistent doses: all volunteers showed significant signs of improvement'	'Where was the hallucinogenic chemical compound administered to depressed patients?'
24.	'Cherry seeds have been brought to the International Space Station. After returning to Earth and being planted, the trees bloomed six years earlier than expected, and some were showing unusual flowers'	‘How many years ago did the cherry trees bloom, the seeds of which had been on the International Space Station?'
25.	‘Time ago, most carrots were purple. It was in the 17th century that Dutch growers chose to select and favor orange carrots, to pay homage to one of the colors of their 'era' flag’	'What color were most carrots up to the 17th century?'
