# Supplementary material for: Task-based activation and resting-state connectivity predict individual differences in semantic capacity for complex semantic knowledge
Source: Commun Biol. 2023 Oct 9;6:1020. doi: 10.1038/s42003-023-05400-1 (PMC10562439; doi:10.1038/s42003-023-05400-1)
Supplement: Supplementary file 3 — Reporting Summary [file 42003_2023_5400_MOESM3_ESM.pdf]

nature portfolio

Corresponding author(s): DBPR COMMSBIO-22-1665B

Last updated by author(s): 29/09/2023

Reporting Summary

Nature Portfolio wishes to improve the reproducibility of the work that we publish. This form provides structure for consistency and transparency in reporting. For further information on Nature Portfolio policies, see our Editorial Policies and the Editorial Policy Checklist.

Statistics

For all statistical analyses, confirm that the following items are present in the figure legend, table legend, main text, or Methods section.

n/a	Confirmed	
☐	☒	The exact sample size (*n*) for each experimental group/condition, given as a discrete number and unit of measurement
☐	☒	A statement on whether measurements were taken from distinct samples or whether the same sample was measured repeatedly
☐	☒	The statistical test(s) used AND whether they are one- or two-sided<br>*Only common tests should be described solely by name; describe more complex techniques in the Methods section.*
☐	☒	A description of all covariates tested
☐	☒	A description of any assumptions or corrections, such as tests of normality and adjustment for multiple comparisons
☐	☒	A full description of the statistical parameters including central tendency (e.g. means) or other basic estimates (e.g. regression coefficient) AND variation (e.g. standard deviation) or associated estimates of uncertainty (e.g. confidence intervals)
☐	☒	For null hypothesis testing, the test statistic (e.g. *F*, *t*, *r*) with confidence intervals, effect sizes, degrees of freedom and *P* value noted<br>*Give P values as exact values whenever suitable.*
☒	☐	For Bayesian analysis, information on the choice of priors and Markov chain Monte Carlo settings
☒	☐	For hierarchical and complex designs, identification of the appropriate level for tests and full reporting of outcomes
☐	☒	Estimates of effect sizes (e.g. Cohen's *d*, Pearson's *r*), indicating how they were calculated

Our web collection on statistics for biologists contains articles on many of the points above.

Software and code

Policy information about availability of computer code

Data collection	Stimuli were presented and data were collected using Matlab v2017a (www.mathworks.com) and Psychtoolbox Version 3 (www.psychtoolbox.org).
Data analysis	Data were analysed and preprocessed with SPM12 (http://www.fil.ion.ucl.ac.uk/spm/).<br>Data were analysed using the CONN toolbox for Matlab v17a (Whitfield-Gabrieli and Nieto-Castanon, 2012; available at https://www.nitrc.org/projects/conn).<br>The Matlab toolbox MarsBar (Brett et al, 2002) was also used for analyses.

For manuscripts utilizing custom algorithms or software that are central to the research but not yet described in published literature, software must be made available to editors and reviewers. We strongly encourage code deposition in a community repository (e.g. GitHub). See the Nature Portfolio guidelines for submitting code & software for further information.

Data

Policy information about availability of data

All manuscripts must include a data availability statement. This statement should provide the following information, where applicable:

- Accession codes, unique identifiers, or web links for publicly available datasets
- A description of any restrictions on data availability
- For clinical datasets or third party data, please ensure that the statement adheres to our policy

The datasets generated during and/or analysed during the current study are available from the corresponding author on reasonable request.

Human research participants

Policy information about studies involving human research participants and Sex and Gender in Research.

Reporting on sex and gender	The final sample included a total of 43 participants: 16 males and 27 females. Sex were defined based on self reporting.<br>Specific information about gender has not been collected.<br>Specific sex- or gender-based analysis was not computed nor reported, because it was beyond the scopes of the present study.
Population characteristics	Seventy-three participants (20 males, mean age = 24.4 years) were pre-screened.<br>The final sample included a total of 43 participants (16 males, mean age = 23.4 years), 41 of which were included in the fMRI task analysis (14 males, mean age = 24.1 years). All selected participants were right-handed native Italian speakers, with no history of neurological disorders.
Recruitment	Participant were recruited through online announcement, mainly using Social Network Platforms (i.e., Facebook, Instagram). Dedicated recruiting channels on that platforms, approved by CIMeC Department (university of Trento), were used. Participants gave informed consent and were reimbursed for participation (15 €/hour for the MRI scanning protocol, 7 €/hour for the behavioural testing)
Ethics oversight	Ethics Committee at the University of Trento

Note that full information on the approval of the study protocol must also be provided in the manuscript.

Field-specific reporting

Please select the one below that is the best fit for your research. If you are not sure, read the appropriate sections before making your selection.

☐ Life sciences ☒ Behavioural & social sciences ☐ Ecological, evolutionary & environmental sciences

For a reference copy of the document with all sections, see nature.com/documents/nr-reporting-summary-flat.pdf

Behavioural & social sciences study design

All studies must disclose on these points even when the disclosure is negative.

Study description	Quantitative experimental study
Research sample	Seventy-three participants (20 males, mean age = 24.4 years) were pre-screened.<br>The final sample included a total of 43 participants (16 males, mean age = 23.4 years), 41 of which were included in the fMRI task analysis (14 males, mean age = 24.1 years)
Sampling strategy	Sampling procedure followed two consecutive steps:<br>1)Pre-screening of 73 participants on the Information and Digit Span subscales of the Wechsler Adult Intelligence Scale 4th Edition (WAIS-IV)<br>2)Selection of participant based on the distribution of data in the aforementioned subscales, in order to have a sample normally distributed in these those measures. Specifically, we randomly selected participants from specific GI normalised scores intervals (6-7;8-9;10-11;12-13;14-15;16-17), thus obtaining a normal distributed sample (43 participants) as mentioned before.
Data collection	Behavioural measures were collected through pen and pencil questionnaires and computer-based experiments, delivered by a single experimenter.<br>Neuroimaging data were acquired at the Center for Mind/Brain Sciences (CIMeC) of the University of Trento,with a Prisma 3T scanner (Siemens AG, Erlangen, Germany) and using a 64-channel head coil. We used custom-made matlab (v2017a) scripts to deliver stimuli and collect behavioural data form task-based fMRI.

Timing	Pre-screening data were collected online between October and November 2020. fMRI data were collected between November 2020 and February 2021.
Data exclusions	Two participants were excluded from the fMRI-task analysis due to within-run head movements exceeding 2.5 mm in two or more runs. Exclusion criteria were pre-enstablished
Non-participation	No participants dropped out/declined participation.
Randomization	Participants were not allocated in experimental groups

Reporting for specific materials, systems and methods

We require information from authors about some types of materials, experimental systems and methods used in many studies. Here, indicate whether each material, system or method listed is relevant to your study. If you are not sure if a list item applies to your research, read the appropriate section before selecting a response.

Materials & experimental systems

n/a	Involved in the study
☒	☐ Antibodies
☒	☐ Eukaryotic cell lines
☒	☐ Palaeontology and archaeology
☒	☐ Animals and other organisms
☒	☐ Clinical data
☒	☐ Dual use research of concern

Methods

n/a	Involved in the study
☒	☐ ChIP-seq
☒	☐ Flow cytometry
☐	☒ MRI-based neuroimaging

Magnetic resonance imaging

Experimental design

Design type	Task-based fMRI (event-related) and Resting State fMRI
Design specifications	The task-based fMRI session was divided into 4 experimental runs lasting 7 minutes each. In each run, 15 questions for each of the four knowledge-domains and 12 trials of the control condition were presented in a pseudo-randomised event-related design (72 trials in each run). The fMRI task session thus included 288 trials in total: 240 questions (60 for each domain) and 48 control trials. Trial duration was 6 seconds with 2-3 seconds of word presentation (depending on sentence length), followed by a red fixation cross that cued the response interval.<br>We collected also 2 separate block of 8-minutes-long resting state runs.
Behavioral performance measures	We recorder button-press responses (1,2 indicating Known or Unknown facts) for the experimental condition and single button press responses for the control condition. Average response for each condition were considered to ensure that participants were performing the task as expected.

Acquisition

Imaging type(s)	functional (task-based + resting state) and structural images
Field strength	3 Tesla
Sequence & imaging parameters	Functional images were acquired using echo planar (EPI) T2*-weighted scans. Acquisition parameters were: repetition time (TR) of 2 s, an echo time (TE) of 28 ms, a flip angle of 75°, a field of view (FoV) of 100 mm, and a matrix size of 100 x 100. Total functional acquisition consisted of 888 volumes, for the four experimental runs, and 470 volumes for the two 8-minute-long resting state runs, each of 78 axial slices (which covered the whole brain) with a thickness of 2 mm and gap of 2 mm, AC/PC aligned. Two 6-minute-long high resolution (1×1×1 mm) T1-weighted MPRAGE sequences were also collected (sagittal slice orientation, centric phase encoding, image matrix = 288 × 288, field of view = 288 mm, 208 slices with 1-mm thickness, repetition time = 2290, echo time = 2.74, TI = 950 ms, 12° flip angle).
Area of acquisition	Whole brain scan

Diffusion MRI ☐ Used ☒ Not used

Preprocessing

Preprocessing software	Data were analysed and preprocessed with SPM12 (http://www.fil.ion.ucl.ac.uk/spm/) toolbox of Matlab.<br>The first four volumes of each run were dummy scans. All images were slice-time corrected, realigned to correct for head movement, normalised to MNI space and smoothed using a 6 mm FWHM isotropic kernel. Before computing the General Linear Model, the four runs were concatenated to avoid empty parameters in one or more conditions.

Normalization	Linear Normalisation to MNI space
Normalization template	ICBM152
Noise and artifact removal	The six head-motion parameters were included as additional regressors of no interest.
Volume censoring	/

Statistical modeling & inference

Model type and settings	fMRI task: mass univariate analysis were used. Before computing the General Linear Model, the four runs were concatenated to avoid empty parameters in one or more conditions. We had a 2(Response: Known and Unknown) x 4 (Knowledge-Domain: People, Places, Objects, Scholastic) within subject design.<br>Group-level analysis was performed in one random-effects GLM and two separate whole-brain regressions, with the predictor variables Omni-semantic and Omni executive as regressors.
Effect(s) tested	-Known vs. Unknown, ANOVA<br>-To identify knowledge-domain-selective voxels, we considered both known trials and unknown trials and contrasted each single category to the average of the remaining three (e.g. [People > Places, Objects, Scholastic]), ANOVA,<br>-Linear Regression analysis with two distinct regressors (Omni-Semantic, Omni-Executive)

Specify type of analysis: ☐ Whole brain ☐ ROI-based ☒ Both

Anatomical location(s): Probabilistic atlases were used to name the anatomical locations of the activation clusters.

Statistic type for inference (See Eklund et al. 2016)	cluster-wise
Correction	FWE

Models & analysis

n/a	Involved in the study
☐	☒ Functional and/or effective connectivity
☒	☐ Graph analysis
☒	☐ Multivariate modeling or predictive analysis

Functional and/or effective connectivity	Resting-State Functional Connectivity: Functional connective indices (Fisher-transformed Pearson's correlation coefficients) were then calculated between a) regions of interest (ROI) and all voxels and b) ROI to ROI (see results). At the group level, intrinsic connection strengths were assessed via second-level RFX analysis and individual differences were assessed by regressing connectivity indices with the predictors of interest across participants.
